# Association between maternal sugar-sweetened beverage consumption and the social-emotional development of child before 1 year old: A prospective cohort study

**DOI:** 10.3389/fnut.2022.966271

**Published:** 2022-11-18

**Authors:** Rui Gao, Xuemei Liu, Xiuxiu Li, Yuanxing Zhang, Min Wei, Peng Sun, Jianan Zhang, Li Cai

**Affiliations:** ^1^Department of Science and Education, Shenzhen Birth Cohort Study Center, Nanshan Maternity and Child Healthcare Hospital of Shenzhen, Shenzhen, China; ^2^Shenzhen Cadre and Talent Health Institute (Shenzhen Talent Institute), Shenzhen, China; ^3^Department of Maternal and Child Health, School of Public Health, Sun Yat-sen University, Guangzhou, China; ^4^Guangdong Provincial Key Laboratory of Food, Nutrition and Health, School of Public Health, Sun Yat-sen University, Guangzhou, China

**Keywords:** sugar-sweetened beverages (SSB), social-emotional functioning/development, birth cohort study, pregnancy, ASQ-SE

## Abstract

**Background:**

Excessive consumption of sugar-sweetened beverages (SSBs) has become an international public health issue. Adverse effects of sugary beverage consumption on both mother and child during pregnancy continue to be found. However, evidence regarding maternal SSB consumption and social-emotional development of children is lacking.

**Methods:**

Based on the Shenzhen Birth Cohort Study (loss rate: 10.97%), we included 985 mother-infant pairs from 2018 to 2022. All mothers had a singleton live birth without hypertension, diabetes, tumor, or serious immune system disease before pregnancy. We used a chart of frequency distribution to show maternal SSB consumption, including non-diet soda, tea drinks (not 100% tea), fruit drinks, Sugar-sweetened coffee, bubble tea, or cocoa drinks, and total SSBs. A multivariate logistic regression model was used to estimate the odds ratios of the potential delay on social-emotional development of each child was monitored at both 6 months and 12 months of age based on maternal SSB consumption.

**Results:**

Among the mothers, 728 (73.91%) drank SSBs <1 time per week, 194 (19.70%) drank SSBs 1–2 times per week, 43 (4.37%) drank SSBs 3–4 times per week, and 20 (2.03%) drank SSBs 5 or more times per week. Children aged 12 months with mothers who drank SSBs five or more times per week during pregnancy had an increased risk of potential delay on social-emotional development compared to those with mothers who drank SSBs less than once per week [odds ratio: 3.08 (1.13–8.39)]. Regarding the specific kinds of SSBs, we found that tea drinks (not 100% tea) were positively associated with potential delay on social-emotional development in children aged 6 months.

**Conclusion:**

Nearly three-quarters of mothers consumed almost no SSBs during pregnancy. High SSB intake during pregnancy was associated with an increased risk of the potential delay on social-emotional development of a child at 6 and 12 months of age.

## Introduction

Sugar-sweetened beverages (SSBs) refer to beverages with added artificial sugar or beverages with more than 5% of added sugar content (1), according to the Food Guide Pagoda for Chinese Residents (2022). SSBs have become an international public health issue since their intake frequency and portion size have increased (2, 3). In China, SSBs were ranked in the top three contributors of total fluid intake (TFI). The median daily intake of SSBs in adult females (18–55 years) was reported as 163 mL/day (4). The contribution of water intake from SSBs among Chinese pregnant women in 2018 accounted for 1.4% (5). Many reports have revealed the role of SSBs in obesity, cardiovascular disease, type 2 diabetes, and chronic diseases (6–8). There is also evidence that the negative consequences of soft drinks can be linked to cognitive impairment, such as stroke and dementia, oxidative stress, and poorer sleep quality and duration of sleep (9, 10). Potential mechanisms include an increase in glutathione-6-dehydrogenase levels, increased levels of gamma-aminobutyric acid (GABA), and glutamate and dopamine alteration in brain waves using electroencephalography (EEG) (10).

During pregnancy, sugar-sweetened beverage intake was also associated with an increased risk of pre-eclampsia, preterm delivery, and other pregnancy complications (11). In addition, accumulating evidence has linked SSB intake during pregnancy to birth size, allergic disease, obesity risk, and other developmental problems of offspring in later life (12–16). Further, Berger et al. revealed that infant neurodevelopmental outcomes at 24 postnatal months can be adversely affected by maternal fructose intake in early lactation (17).

However, there seem to be no human studies describing the relationship between prenatal SSB intake and cognitive function development of offspring. Social emotion is a representative part of neurobehavioral development. Previous reports suggested that children who received social-emotional intervention had better academic achievements and lower emotional stress (18). These children also showed better self-attitude, behavior, and ability to manage stress (19). Since China is a populous country, this has become a question worthy of further study. According to China's national census data, the population growth of Shenzhen ranks first in China, in which the fertility policy has played a continuous and effective role.

In this study, we describe maternal SSB consumption in Shenzhen, China and explore the relationship between maternal SSB consumption and the social-emotional development of child before 1 year old.

## Methods

### Participants

Mother-infant pairs were recruited from Shenzhen Birth Cohort Study (SZBC, NCT03830879), a population-based prospective cohort study at Nanshan Maternity and Child Healthcare Hospital of Shenzhen. SZBC was designed to identify early environmental and genetic causes of normal and abnormal growth, development, and health from fetal life until young adulthood. Briefly, pregnant women were recruited by SZBC staff at the first antenatal visit around gestational weeks 6–12. Enrolled participants completed three face-to-face interviews in all three trimesters during pregnancy. When the children were born, a serious of follow-up interviews including questionnaires, bio-sample collecting, and developmental assessment were carried out with the mother and child.

In this study, we included all mothers with a live singleton birth, and whose SSB information during pregnancy and Ages & Stages Questionnaire: Social-Emotional (ASQ-SE) for the child at 6 months and 12 months of age was available, and we excluded mothers with hypertension, diabetes, tumor, and serious immune system disease before pregnancy. Our study finally consisted of 985 mother-infant pairs. This study followed the guidelines for reporting observational studies—Strengthening the Reporting of Observational Studies in Epidemiology (STROBE) statement (Supplemental materials 1)—and were approved by ethics committees at Nanshan Maternity & Child Healthcare Hospital of Shenzhen (NSFYEC-KY-2020031) and Sun Yat-sen university (2018-054).

### Exposure: Maternal SSB intake during pregnancy

Considering possible loss of appetite, nausea, and vomiting in the first trimester, the second trimester was selected to collect dietary intake information. Information on maternal SSBs was collected using a self-administered questionnaire between the 20th and 28th gestational week. Food photographs with standard portions sizes were provided to the mothers. SSBs consisted of non-diet soda, fruit drinks (not 100% fruit juice), sugar-sweetened coffee, bubble tea, or cocoa drink, and tea drinks (not 100% tea). Mothers needed to answer how often they had drunk SSBs in the past month (< 1 time per week, 1–2 times per week, 3–4 times per week, or 5 or more times per week).

### Outcome: Social-emotional development of child

Child social-emotional development was measured by the Chinese version of the ASQ-SE, based on children at 6 months and 12 months of age. The ASQ-SE is a screening tool to capture the potential delay of child social-emotional development and assesses self-regulation, compliance, social-communication, adaptive functioning, autonomy, affect, and interaction with people. The score was assigned according to the answers provided by the caregiver, and lower scores indicate better social-emotional development in the child. The total score ranges from 0 to 120 for children at 6 months, and 0 to 160 for children at 12 months. According to the ASQ-SE instruction (20), the potential delay on social-emotional development at 6 or 12 months of age was defined as a total score of ≥40. Cronbach's alpha coefficient was 0.8 for the Chinese version of ASQ-SE, and sensitivity and specificity were 87.50 and 84.48%, respectively (21).

### Covariates

Covariates were selected according to previous studies and preliminary studies of our research, including socio-demographics, environmental factors during pregnancy, obstetric conditions, and child feeding. Information on mother's age (< 25 years old, 25–34 years old, ≥35 years old), education level (lower than college, college, higher than college), monthly income level (<5,000 yuan, 5,000–10,000 yuan, 10,000–15,000 yuan, 15,000–20,000 yuan, ≥20,000 yuan), parity (0, ≥1), smoking or drinking before pregnancy (yes, no), and initiation of prenatal care was collected by SZBC First Trimester Questionnaire when the study participants joined the cohort before their 19th gestational week. Information on threatened abortion (yes, no), preterm birth (yes, no), and maternal complications (yes, no)—which were defined as gestational diabetes, gestational hypertension, hyperthyroidism, or hypothyroidism—was collected directly from mothers' medical records. Information on breastfeeding more than 6 months (yes, no) and initiation of complementary feeding was collected by the SZBC Child Questionnaire at 12 months.

### Statistical analysis

We assessed the distribution of sociodemographic and maternal characteristics according to maternal SSB consumption. Comparisons between categorical variables were tested using chi-square tests. We used chart of frequency distribution to show maternal SSB consumption. Mothers were divided into four groups based on how often they drank SSBs, <1 time per week, 1–2 times per week, 3–4 times per week, and 5 or more times per week.

Multivariate logistic regression models were used to estimate the odds ratios (ORs) and 95% CI of the potential delay on social-emotional development of the child at 6 and 12 months of age based on maternal SSB consumption. Covariates listed above were adjusted in these models. The <1 time per week group was used as the reference group when maternal SSB consumption was evaluated as a rank variable in these models.

We also performed a sensitivity analysis by taking four kinds of SSBs (non-diet soda, tea drinks (not 100% tea), fruit drinks (not 100% fruit juice), and sugar-sweetened coffee, bubble tea, or cocoa drinks) as continuous variables into multivariate logistic regression models.

Two-sided *P* < 0.05 were considered statistically significant. All statistical analyses were conducted using survey modules of SAS software version 9.4 (SAS Institute, Cary, North Carolina).

## Results

This study comprised 985 mothers-child pairs. A total of 13.71% (*n* = 135) and 15.74% (*n* = 155) children showed potential delay in social-emotional development at 6 months and 12 months, respectively. Among the mothers, 728 (73.91%) drank SSBs <1 time per week, 194 (19.70%) drank SSBs 1–2 times per week, 43 (4.37%) drank SSBs 3–4 times per week, and 20 (2.03%) drank SSBs 5 or more times per week. Table 1 presents a comparison of socio-demographics, environmental factors during pregnancy, obstetric conditions, and child-feeding characteristics of the mothers in four SSB groups. Women who were younger or had complications drank SSBs more often than their counterparts (Table 1).

**Table 1:** Characteristics of the study population, according to maternal total sugar-sweetened beverage consumption.

**Variables**	***N***	**Total sugar-sweetened beverage consumption**			
		**<1 time per week**	**1–2 times per week**	**3–4 times per week**	**5 or more times per week**
Overall	985 (100%)	728 (73.91%)	194 (19.70%)	43 (4.37%)	20 (2.03%)2.0 (.03%)
Age, years, *n* (%)^a^					
<25	54 (5.48%)	33 (4.53%)	14 (7.22%)	4 (9.30%)	3 (15.00%)
25–34	731 (74.21%)	530 (72.80%)	155 (79.90%)	32 (74.42%)	14 (70.00%)
≥35	200 (20.30%)	165 (22.66%)	25 (12.89%)	7 (16.28%)	3 (15.00%)
Education levels, *n* (%)					
Lower than college	397 (40.30%)	287 (39.42%)	88 (45.36%)	15 (34.88%)	7 (35.00%)
College	483 (49.04%)	365 (50.14%)	88 (45.36%)	21 (48.84%)	9 (45.00%)
Higher than college	105 (10.66%)	76 (10.44%)	18 (9.28%)	7 (16.28%)	4 (20.00%)
Income (RMB), *n* (%)					
<5,000	169 (17.16%)	119 (16.35%)	36 (18.56%)	8 (18.60%)	6 (30.00%)
5,000–9,999	460 (46.70%)	342 (46.98%)	92 (47.42%)	19 (44.19%)	7 (35.00%)
10,000–14,999	206 (20.91%)	150 (20.60%)	45 (23.20%)	9 (20.93%)	2 (10.00%)
15,000–19,999	80 (8.12%)	67 (9.20%)	9 (4.64%)	2 (4.65%)	2 (10.00%)
≥20,000	70 (7.11%)	50 (6.87%)	12 (6.19%)	5 (11.63%)	3 (15.00%)
Parity, *n* (%)					
0	561 (56.95%)	410 (56.32%)	116 (59.79%)	23 (53.49%)	12(60.00%)
≥1	424 (43.05%)	318 (43.68%)	78 (40.21%)	20 (46.51%)	8 (40.00%)
Smoking or drinking before pregnancy, *n* (%)^a^					
Yes	279 (28.32%)	190 (26.10%)	70 (36.08%)	11 (25.58%)	8 (40.00%)
No	706 (71.68%)	538 (73.90%)	124 (63.92%)	32 (74.42%)	12 (60.00%)
Pregnancy complications, *n* (%)					
Yes	471 (47.82%)	344 (47.25%)	94 (48.45%)	23 (53.49%)	10 (50.00%)
No	514 (52.18%)	384 (52.75%)	100 (51.55%)	20 (46.51%)	10 (50.00%)
Threatened abortion, *n* (%)					
Yes	254 (25.79%)	194 (26.65%)	52 (26.80%)	7 (16.28%)	1 (5.00%)
No	731 (74.21%)	534 (73.35%)	142 (73.20%)	36 (83.72%)	19 (95.00%)
Preterm birth, *n* (%)					
Yes	35 (3.55%)	23 (3.16%)	10 (5.15%)	1 (2.33%)	1 (5.00%)
No	950 (96.45%)	705 (96.84%)	184 (94.85%)	42 (97.67%)	19 (95.00%)
Breastfeeding more than 6 months, *n* (%)					
Yes	793(80.51%)	600 (82.42%)	144 (74.23%)	33 (76.74%)	16 (80.00%)
No	192(19.49%)	128 (17.58%)	50(25.77%)	10(23.26%)	4 (20.00%)
Initiation of prenatal care, mean ± SD					
Weeks	5.46 ± 0.69	5.49 ± 0.68	5.37 ± 0.73	5.37 ± 0.66	5.25 ± 0.64
Potential delay on social-emotional development at 6 months, *n* (%)					
Yes	135 (13.71%)	93 (12.77%)	36 (18.56%)	3 (6.98%)	3 (15.00%)
No	850 (86.29%)	635(87.23%)	158(81.44%)	40 (93.02%)	17 (85.00%)
Potential delay on social-emotional development at 12 months, *n* (%)					
Yes	155 (15.74%)	107 (14.70%)	35 (18.04%)	6 (13.95%)	7 (35.00%)
No	830 (84.26%)	621 (85.30%)	159 (81.96%)	37 (86.05%)	13 (65.00%)

a P < 0.05.

The percentage in brackets was the composition ratio of each horizontal item.

**Figure 1:**
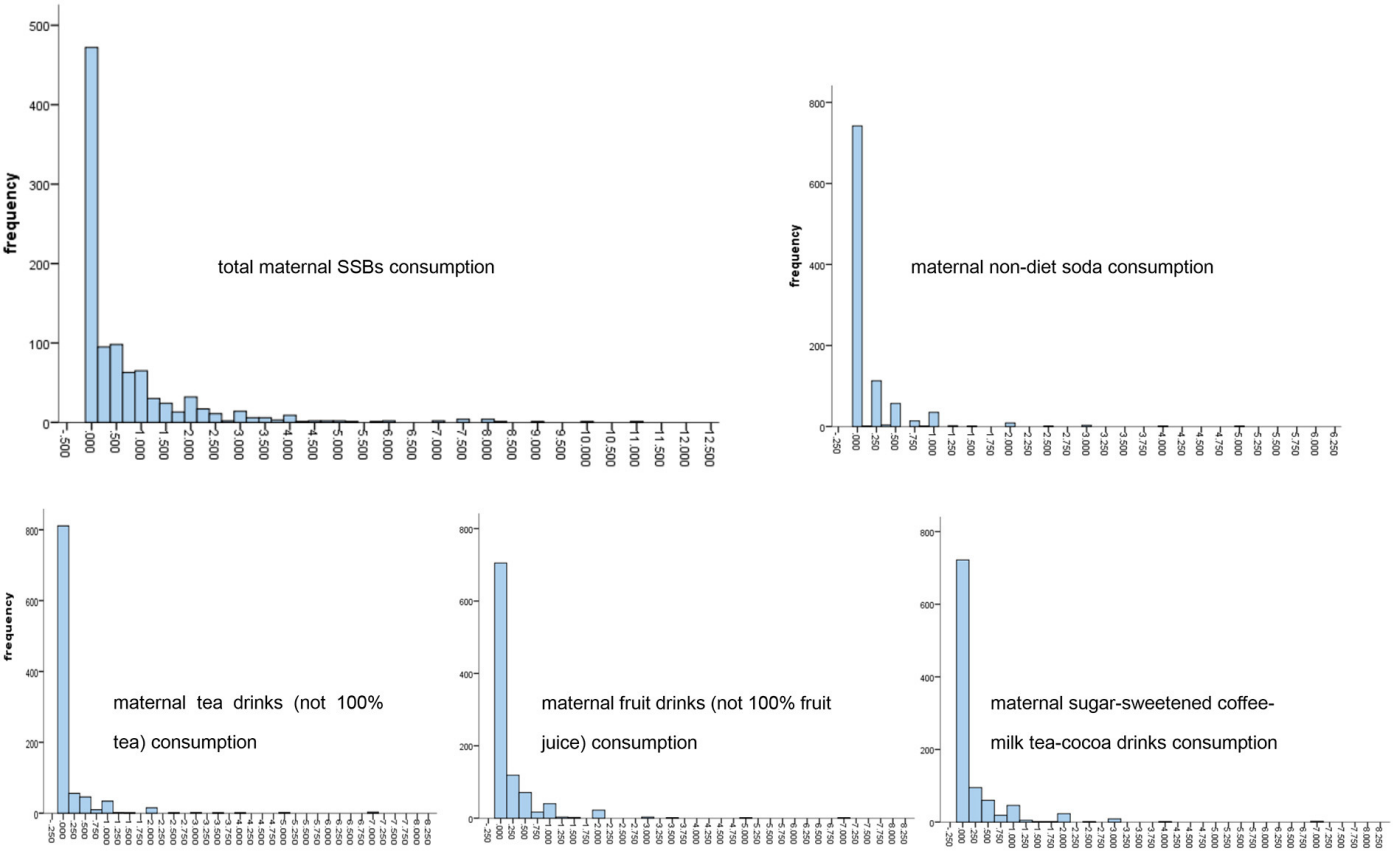
Frequency distribution of maternal SSBs consumptions.

There were L-shaped frequency distributions on the consumption of total SSBs and four kinds of SSBs, non-diet soda, tea drinks (not 100% tea), fruit drinks (not 100% fruit juice), and sugar-sweetened coffee, bubble tea, cocoa drinks during pregnancy. In our research, many mothers consumed almost no SSBs during pregnancy. The frequencies of non-diet soda were 94.62, 4.87, 0.41, and 0.10% in the < 1 time per week group, 1–2 times per week group, 3–4 times per week group, and 5 or more times per week group, respectively; while the frequencies of tea drinks (not 100% tea) were 93.71, 5.38, 0.40, and 0.51%; of fruit drinks (not 100% fruit juice) were 92.59, 6.80, 0.41, and 0.20%; and of sugar-sweetened coffee, milk, bubble tea, cocoa drinks were 90.96, 7.82, 1.02, and 0.20% in the four groups, respectively (Figure 1).

By using the multivariate logistic regression, we found that mothers who drank SSBs five or more times per week during pregnancy had increased odds of potential delay on the social-emotional development of their child at 12 months of age compared to mothers who drank SSBs <1 time per week. The adjusted OR was 3.08 (95% CI, 1.13–8.39) (Table 2).

**Table 2:** Associations between maternal total sugar-sweetened beverage consumption and social-emotional development at child at 6 months and 12 months of age.

		**Potential delay on social-emotional development (ASQ-SE** ≥**40)**	
		**6-month OR (95%CI)**	**12-month OR (95%CI)**
Total sugar-sweetened beverages consumption ^a^		1.02 (0.88–1.17)	1.11 (0.98–1.25)
Total sugar-sweetened beverages consumption ^b^	<1 time per week	1.00	1.00
	1–2 times per week	1.50 (0.97–2.33)	1.15 (0.74–1.78)
	3–4 times per week	0.53 (0.16–1.78)	0.91 (0.37–2.25)
	5 or more times per week	1.26 (0.34–4.65)	**3.08 (1.13**–**8.39)**

a Evaluated as a continuous variable.

b Evaluated as a rank variable, and <1 time per week group was used as the reference group.

Age, education, income, parity, smoking or drinking before pregnancy, pregnancy complications (e.g., gestational diabetes, gestational hypertension, hyperthyroidism, hypothyroidism), threatened abortion, preterm birth, breastfeeding more than 6 months, and initiation of complementary food were adjusted in models.

The bold values indicate that the ORs are statistically different.

As shown in Table 3, there was a positive association between the consumption of tea drinks (not 100% tea) during pregnancy and the social-emotional development of the child at 6 months of age [OR:1.39 (95% CI, 1.06–1.82)]. The frequency of drinking different kinds of SSBs during pregnancy had a nonsignificant relationship with children's social-emotional development at 12 months of age, with the ORs ranging from 1.03 to 1.27.

**Table 3:** Associations of four kinds of maternal sugar-sweetened beverages consumption on social-emotional development at child at 6 and 12 months.

**Specific kinds of sugar-sweetened beverages**	**Potential delay on social-emotional development**	
	**6-month OR (95%CI)**	**12-month OR (95%CI)**
Non-diet soda	1.23 (0.77–1.96)	1.10 (0.72–1.68)
Tea drinks (not 100% tea)	**1.39 (1.06**–**1.82)**	1.03 (0.77–1.37)
Fruit drinks (not 100% fruit juice)	0.64 (0.38–1.08)	1.27 (0.93–1.73)
Sugar-sweetened coffee, bubble tea, cocoa drinks	0.82 (0.54–1.26)	1.06 (0.79–1.43)

Age, education, income, parity, smoking or drinking before pregnancy, pregnancy complications (e.g., gestational diabetes, gestational hypertension, hyperthyroidism, hypothyroidism), threatened abortion, preterm birth, breastfeeding more than six months, and initiation of complementary food were adjusted in models.

The bold values indicate that the ORs are statistically different.

## Discussion

With data from SZBC, we found 73.91% women in China drink SSBs <1 time per week, especially soda, during pregnancy. There might be a link between high maternal SSB consumption and a potential delay on social-emotional development of a child at 6 and 12 months of age.

Previous studies found that 49.3% of US adults consumed ≥ 1 SSBs on a given day (2011–2014) (12), with a total of 21.9 and 9.7% of pregnant women consuming SSBs ≥1 time/day and ≥2 times/day, respectively (2017) (22). In China, 47% of adults consumed ≥1 servings of SSB/day (2016), and SSBs were the third most common type of water intake during pregnancy (2018) (9, 17). However, our study showed that nearly three-quarters of mothers consumed SSBs < 1 time/day during pregnancy. The habits of SSB consumption by these mothers are more in line with the Dietary Guidelines for Chinese Residents (2022). The potential causes for the difference may be that the mothers in our study had higher education levels (60% college and above) and are more likely to have received systematic and comprehensive education about healthy diets (17, 23).

To the best of our knowledge, this is the first study regarding the association between maternal sugar-sweetened beverage intake and the social-emotional development of children. A prospective birth cohort study in Boston, US, found that greater prenatal sucrose and SBB intake by mothers were associated with poorer cognition among offspring. However, this study focused on cognition, such as intelligence, memory, and learning, in children aged three to seven years old, and it is difficult to distinguish between pre- and postnatal effects on children neurodevelopmental outcomes, because children's own eating or drinking habits and acquired environmental factors have a great influence on their neurodevelopmental development (9, 24). Overall, our results are consistent with the findings of the study in Boston, which support the varying degrees of adverse associations of maternal SSB consumption on child development.

Although the detailed mechanisms underlying the association between maternal sugar-sweetened beverage intake and social-emotional development remain to be understood, placental and fetal growth and metabolism are thought to be the leading factors (12). An animal study revealed that a maternal high-sugar diet can disrupt the N-methyl-D-aspartic acid (NMDA) receptor composition and regulation in the medial prefrontal cortex and the hippocampus, which might influence the social-emotional function of offspring (25). Further research found that fructose, the content of SSBs, was partly responsible for cognitive damage (14).

This study has important clinical and public health implications. The increasing consumption of SSBs is a worldwide problem. The low intake of SSBs for the majority of mothers in our study might be due to the positive impact of promotion of various nutrition and health programs. Researchers found that dietary intervention for adolescents combined with dietary guidelines for Americans has achieved certain favorable results in the US in recent years (13). How to combine the latest version of Dietary Guidelines for Chinese residents with health education and health intervention during pregnancy is worth further discussion. Furthermore, we found that maternal SSB consumption poses a risk to the potential delay of social-emotional development of children under 1 year old despite the low overall intake of SSBs in this population, which could explain the value of nutrition and health projects from another aspect. This finding can also help physicians who provide preconception or prenatal care. According to the latest Dietary Guidelines for Chinese Residents, adults are advised to drink fewer or no SSBs. Our study found that more than 25% of pregnant women consume one serving or more of SSBs per week, indicating more efforts are needed from different aspects, including governments, medical care institutions, the food industry, community groups, media, and individuals.

This study benefits from using longitudinal statistical modeling of data drawn from a prospective Shenzhen birth cohort study. However, there are several limitations to note. First, we only focused on the frequency of beverage intake and not on the amount consumed. Second, at lower intake of SSBs, we did not have enough power to perform stratified analyses, such as by age group. Third, there was recall bias in the survey of SSB intake. However, we tried to reduce the bias by providing food photographs with standard portion sizes, one-to-one surveys, and other quality control methods. Fourth, we could not include all potential covariates associated with children's outcomes, leading to potential for residual or unmeasured confounding bias. Lastly, the findings should be interpreted conservatively because of the observational nature of the study.

## Conclusions

In this population-based prospective birth cohort study, we found nearly three-quarters of mothers consume almost no SSBs during pregnancy. High SSB intake during pregnancy was associated with an increased risk of potential delay to the social-emotional development of children aged 6 and 12 months. Further investigation is needed to understand the underlying mechanisms.

## Data availability statement

The raw data supporting the conclusions of this article will be made available by the authors, without undue reservation.

## Ethics statement

The studies involving human participants were reviewed and approved by Nanshan Maternity and Child Healthcare Hospital in Shenzhen, China, Sun Yat-sen University, Guangzhou, China. Written informed consent to participate in this study was provided by the participants' legal guardian/next of kin.

## Author contributions

RG and LC conceived and designed the study. XLiu and RG conducted the statistical analysis and drafted the manuscript. XLi supervised the study and provided technical support. All authors acquired, analyzed, or interpreted the data, and critically revised the article for important intellectual content. All authors contributed to the article and approved the submitted version.

## Funding

Medical Scientific Research Foundation of Guangdong Province of China (A2021123); the Science and Technology Planning Project of Shenzhen Nanshan District (2020032 General); and the Sanming Project of Medicine in Shenzhen (SZSM201803061). The funders had no role in the design and conduct of the study; collection, management, analysis, and interpretation of the data; preparation, review, or approval of the manuscript; and decision to submit the manuscript for publication.

## Conflict of interest

The authors declare that the research was conducted in the absence of any commercial or financial relationships that could be construed as a potential conflict of interest.

## Publisher's note

All claims expressed in this article are solely those of the authors and do not necessarily represent those of their affiliated organizations, or those of the publisher, the editors and the reviewers. Any product that may be evaluated in this article, or claim that may be made by its manufacturer, is not guaranteed or endorsed by the publisher.
