# Supplementary material for: Association between maternal sugar-sweetened beverage consumption and the social-emotional development of child before 1 year old: A prospective cohort study
Source: Front Nutr. 2022 Nov 18;9:966271. doi: 10.3389/fnut.2022.966271 (PMC9716068; doi:10.3389/fnut.2022.966271)
Supplement: Supplementary file 1 [file Table_1.DOCX]

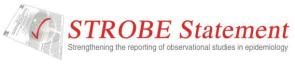


STROBE Statement—Items to be included when reporting observational studies in a conference abstract

**Item** **Recommendation**

| Title | Page 1lines 1-3. Indicate the study’s design with a commonly used term in the title (e.g cohort, case- control, cross sectional) |
| --- | --- |
| Authors | Page 1 lines 5-6. Contact details for the corresponding author |
| Study design | Page 2 lines 28-29. Description of the study design (e.g cohort, case-control, cross sectional) |
| Objective | Page 5 lines 103-105. Specific objectives or hypothesis |
| Methods | |
| Setting | Page 2 lines 28-29. Description of setting, follow-up dates or dates at which the outcome events occurred or at which the outcomes were present, as well as any points or ranges on other time scales for the outcomes (e.g., prevalence at age 18, 1998-2007). |
| Participants | Page 6 lines 109-118. *Cohort* *study*—Give the most important eligibility criteria, and the most important sources and methods of selection of participants. Describe briefly the methods of follow-up |
| Variables | Page 7-8 lines 140-159. Clearly define primary outcome for this report. |
| Statistical  methods | Page 8-9 lines 177-199. Describe statistical methods, including those used to control for confounding |
| Results | |
| Participants | Page 15 lines 263. Report Number of participants at the beginning and end of the study |
| Main results | Page 15-16 lines 272-298. Report estimates of associations. If relevant, consider translating estimates of relative risk into absolute risk for a meaningful time period  Report appropriate measures of variability and uncertainty (e.g., odds ratios with  confidence intervals |

Conclusions Page 21 lines 391-396. General interpretation of study results

STROBE_checklist_conference_abstract_DRAFT_v03 1
